# Effects of Minimally Processed Red Meat within a Plant-Forward Diet on Biomarkers of Physical and Cognitive Aging: A Randomized Controlled Crossover Feeding Trial

**DOI:** 10.1016/j.cdnut.2025.107615

**Published:** 2025-12-10

**Authors:** Saba Vaezi, Bruna O de Vargas, Lee Weidauer, Jessica L Freeling, Moul Dey

**Affiliations:** 1 School of Health and Human Sciences, South Dakota State University, Brookings, SD, United States; 2 FreelingBio Research Consulting, LLC, Vermillion, SD, United States

**Keywords:** minimally processed lean red meat, plant-forward diet, aging, physical function, cardiometabolic health, feeding study, cognitive decline

## Abstract

**Background:**

Popular dietary patterns for cardiovascular and cognitive health often emphasize limiting red meat intake. However, evidence specifically examining the effects of minimally processed lean red meat, independent of processed varieties, remains limited.

**Objectives:**

This study aimed to evaluate the impact of incorporating minimally processed lean red meat into a plant-forward dietary pattern aligned with the 2020–2025 Dietary Guidelines for Americans on markers of age-related cognitive, metabolic, and physical health.

**Methods:**

This 18-wk randomized controlled crossover feeding trial tested a red meat diet with 162 g/d minimally processed pork (MPP) against a macronutrient- and energy-matched no-meat control diet with minimally processed lentils (MPL) in ≥65 y older adults. Metabolic biomarkers related to cognitive and physical health were explored. Primary and secondary endpoints comprised 5 cardiovascular markers, 12 nutritional and neurotransmitter measures, and 2 metrics each of body composition and muscular fitness. Data were analyzed with robust mixed-effects models adjusted for covariates.

**Results:**

Thirty-six Midwestern older adults (26/10 females/males; mean age 71.7 y; mean body mass index: 28 kg/m^2^) completed the study. Cognitive-related metabolic biomarkers improved across both arms. Adoption of the plant-forward diet led to a reduction in fasting insulin after both MPP and MPL phases (*P* < 0.001), and Single Point Insulin Sensitivity Estimator Index increased after MPP (*P* = 0.032), with no significant between-diet differences. High-density lipoprotein concentrations were higher post-MPP than post-MPL (*P* = 0.034). Body weight decreased in both phases (*P* < 0.05), with a trend toward smaller lean mass loss post-MPP. Grip strength and chair-rise performance were maintained throughout the intervention. Neuroactive metabolites and bioactive amino acid concentrations shifted favorably after both interventions.

**Conclusions:**

Findings challenge the perception that red meat is broadly unsuitable for older adults. Including familiar foods like red meat (pork), particularly in minimally processed form and within a healthy overall dietary pattern, may provide age-associated health benefits and improve adherence to plant-forward diets in populations where red meat remains popular.

This trial was registered at clinicaltrials.gov as NCT05581953 (12 October, 2022) and NCT06261775 (7 February, 2024).

## Graphical abstract

**Figure:**
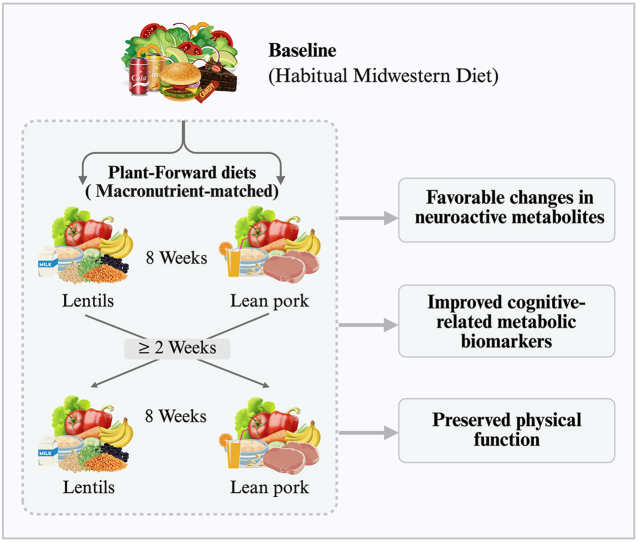

## Introduction

The United States population is rapidly aging, creating a demographic shift and increased healthcare burden due to age-related chronic diseases. Major health challenges associated with aging include cardiovascular diseases (CVD), sarcopenia-related loss of physical function, and cognitive decline. Nearly a quarter of community-dwelling Americans aged 65 y and older have poor health, with an additional 1.3 million living in nursing homes, unable to maintain independence [[1], [2], [3], [4]]. Lifestyle approaches have the potential to delay the onset of aging-associated health decline and loss of independence, thereby improving quality of life and healthspan in older adults [5,6].

Among age-related conditions, cognitive impairment, particularly dementia, poses a significant and growing concern. Dementia is a progressive neurodegenerative disorder characterized by cognitive decline and loss of functional independence, with Alzheimer’s disease (AD) being the most common form [7]. With limited options for early diagnosis, prevention, and treatment, ∼14 million older Americans are projected to have dementia by 2060 [8]. This underscores the urgent need for strategies that target modifiable dementia risk factors.

Emerging evidence suggests that, in addition to being a major contributor to CVD, metabolic dysfunction may play a key role in the development of both cognitive and physical decline in older adults [9,10]. Risk factors such as insulin resistance (IR) and obesity are strongly associated with age-related cognitive decline [11]. IR has received particular attention, as impaired brain glucose metabolism may precede clinical dementia symptoms by over a decade [12]. Due to this pronounced link, AD has been referred to as "type 3 diabetes" [13]. In addition, because the anabolic role of insulin promotes muscle protein synthesis and glucose uptake, IR in aging is also closely linked to muscle loss, sarcopenia, and loss of physical strength [14].

Given these interconnections, lifestyle interventions that target metabolic health hold great promise for preserving both cognitive and physical function in older adults. Diet, physical activity, sleep, and social engagement are increasingly recognized as key determinants of dementia risk [15]. Among these, nutrition stands out for its influence on cognitive-related metabolic health [16,17]. Diets rich in plant-based foods and healthy fats, like the Mediterranean, Dietary Approaches to Stop Hypertension (DASH), and Mediterranean-DASH Intervention for Neurodegenerative Delay diets, have been associated with reduced cognitive decline [[18], [19], [20], [21]]. Although these diets show promise, the use of both processed and unprocessed forms of red meat in research, along with mixed results across studies, points to the need for a deeper understanding of how red meat affects cognitive-related metabolic health. For example, a cohort study reported that replacing a daily serving of processed red meat with legumes reduced dementia risk by 19% [22], and that the consumption of meat is a risk factor for IR and type 2 diabetes across populations [23]. In contrast, a meta-analysis of intervention trials showed that unprocessed red meat intake did not affect weight gain or related metabolic conditions [24], and that red meat can even improve cognitive functionality under certain circumstances [25]. Recent controlled feeding trials further indicate that lean, minimally processed red meat can be integrated into healthy dietary patterns without adverse effects, as demonstrated in our previous study [26]. For example, including lean red meat within a United States-style diet improved lipid profiles and CVD risk factors [27], and in middle-aged and older adults with obesity, a higher-protein hypocaloric diet providing balanced portions of lean beef and pork throughout the day improved lipid profiles, IR scores, and physical function without adverse cardiometabolic effects [28]. Similarly, short-term crossover interventions in overweight females showed that consuming ≤2 servings of lean red meat per day had no adverse effects on glycemic or inflammatory markers, reinforcing that lean red meat can be part of a healthy, nutrient-dense diet [29]. However, randomized controlled trials (RCTs) examining the effects of 100% minimally processed lean red meat on cognitive-related metabolic well-being when consumed as part of healthy dietary patterns in older adults remain scarce.

As such, dietary patterns and active lifestyles that support insulin sensitivity may help reduce cognitive-related metabolic risk in aging populations as well as support physical function. Emerging evidence suggests that diet influences not only conventional nutritional pathways but also neuroactive compounds involved in mood, cognition, and metabolic regulation [30]. Amino acid precursors such as tryptophan and tyrosine contribute to the synthesis of key neuromodulators, including serotonin, dopamine, gamma-aminobutyric acid (GABA), melatonin, and kynurenines [[30], [31], [32], [33]]. Other dietary components may also influence neuromodulator activities indirectly through effects on peripheral metabolism and gut–brain communications [[34], [35], [36], [37]].

We conducted a randomized controlled crossover feeding trial in community-dwelling older adults. Each participant completed two 8-wk dietary intervention phases: one testing a lean red meat diet featuring minimally processed pork (MPP) and the other a macronutrient- and energy-matched control diet based on plant-sourced primary proteins such as lentils, referred to as minimally processed lentil (MPL). A plant-forward dietary pattern emphasizes a primarily plant-based approach with flexibility to include either animal- or plant-sourced proteins. We hypothesized that adding MPP to a nutrient-dense, plant-forward diet aligned with USDA macronutrient recommendations would enhance cognitive-related metabolic health and muscular fitness related to healthspan. Primary outcomes included markers associated with insulin sensitivity, iron stores, and cognitive function along with the chair stand test.

## Methods

This publication reports primary and related secondary outcomes from the Protein-Distinct Macronutrient-Equivalent Diets 2 (PRODMED2) randomized controlled feeding trial. The PRODMED2 trial was registered on 12 October, 2022, at clinicaltrials.gov (NCT05581953) and approved by the Institutional Review Board (IRB) at South Dakota State University (#2209010-EXP). Participant recruitment began shortly after trial registration. Unless otherwise specified, the work was carried out at South Dakota State University in Brookings, South Dakota, in accordance with the Declaration of Helsinki, and all participants provided written informed consent. Detailed information regarding the overall study design and recruitment protocols has been previously published [38]. The present manuscript also reports findings from a separate post hoc study based on the same trial (IRB-2401011-EXM; NCT06261775).

### Study design

This study was a randomized, controlled, 2-arm crossover feeding trial in which all meals were provided to participants in a preportioned, ready-to-heat format to minimize intake variability. The protocol included in-person site visits and data collection at 3 time points: baseline and the end of each dietary phase. Dine-in and food pickup were scheduled in addition to the data collection visits.

### Participants

A total of 36 community-dwelling older adults completed the PRODMED2 feeding trial. Recruitment and screening were conducted between Fall 2022 and Summer 2023 on a rolling basis. Initial eligibility was assessed via telephone screening, and individuals meeting preliminary criteria were invited for an on-site informational and screening visit. At this visit, trained staff conducted clinical measurements and structured interviews to assess health history, medication use, and lifestyle behaviors prior to potential enrollment and informed consent signing.

Eligible participants were adults aged 65 y or older, of any race, sex, education level, or marital status, and generally in good health as confirmed by a routine physical examination within the past year. Inclusion criteria included the absence of medically diagnosed type 2 diabetes, body weight ≥ 110 pounds (∼50 kg), and reporting a habitual omnivorous dietary pattern without special dietary restrictions. Participants were required to consume only study-provided foods (with pork as the sole meat source), attend in-person visits, and abstain from alcohol, supplements, and nonstudy foods for the duration of the trial. Common prescription medication use was allowed, and participants were encouraged to maintain their habitual physical activity patterns throughout the study.

Exclusion criteria included substance use (tobacco and recreational drugs), medications affecting metabolism (e.g., steroids), and diagnoses of major chronic conditions (e.g., cancer, diabetes, history of heart attack or stroke, hepatic or gastrointestinal disease). Participants were also excluded for diagnosed impaired kidney function, recent major surgery that would significantly impair their mobility, mental health concerns affecting compliance, or any reason that would make them unable to attend study visits.

In this RCT, participants were assigned to intervention groups using a 2 × 2 block randomization design (with block sizes of 2 for individuals and 4 for couples). Randomization was conducted manually in a sequentially numbered manner by a study team member not involved in outcome assessments or later data analysis. Participants were assigned to either MPP or MPL diets after randomization. Laboratory personnel responsible for processing biospecimens and collecting assay data were blinded to ID assignments.

### Study outcomes

The primary outcomes of the PRODMED2 trial were serum ferritin, homocysteine, insulin, and chair stand performance. Secondary outcomes included body weight, glucose, triglyceride, total cholesterol (TC), HDL cholesterol, phosphatidylcholine, and grip strength normalized to body mass. Brain-derived neurotrophic factor (BDNF) and a panel of neuroactive and methylation-related metabolites were also assessed as exploratory outcomes to further support primary and secondary observations. These included phenylalanine, glycine, glutamic acid, tyrosine, kynurenine, GABA, tryptophan, and choline. The Single Point Insulin Sensitivity Estimator Index (SPISE) was calculated based on collected data. All outcomes were selected for their relevance to cognitive-related metabolic indicators in older adults and their known responsiveness to short-term dietary interventions [39]. No serious adverse events or potential harm were reported during either diet.

### Dietary intervention and adherence

The diets were designed to align with the 2020–2025 USDA Dietary Guidelines for Americans (DGA) for adults aged 51 and older for macronutrient distribution with a plant-forward emphasis. Every main meal (breakfast, lunch, and dinner) included plant foods providing a mean of 102 servings of plant foods (vegetables, fruits, grains) per week. A moderate amount of dairy, eggs, and plant oils was included. Both diets were implemented in a fully controlled, all-food-provided format, using a 7-d rotating menu developed and analyzed in Nutritionist Pro software (Axxya Systems) to meet target nutrient goals and DGA recommendations. A sample 1-d menu is provided in Supplementary Table 1. As primary protein sources, contributing > 45% of total protein, in 1 arm, participants consumed 5.7 oz/d (162 g/d) of minimally processed lean pork prepared using basic handling steps and simple cooking methods, including fat trimming and roasting in a rotisserie-style oven with only olive oil spray and salt, which allowed excess fat to drain naturally during cooking [40]. In the other arm, participants consumed an equivalent amount of protein from lentils, chickpeas, black beans, and split peas (331.6 g/d, cooked), prepared from whole or simply processed ingredients for 8 wk, separated by a 2 wk washout period. For simplicity, all MPL protein sources are hereafter collectively referred to as “lentils.” To minimize confounding, alcohol, soy, beef, poultry, seafood, and artificial sweeteners were excluded. Each diet intervention was for 8 wk, separated by a 2 wk washout period.

All meals and snacks were prepared by trained ServeSafe-certified staff, portioned to the nearest gram, and provided either on-site or as take-home packages with clear heating instructions. Participants’ baseline diet reflected a typical omnivorous pattern, confirmed via 24-h dietary recalls.

At the end of each dietary phase, participants completed a structured questionnaire assessing adherence, menu acceptability, and overall feasibility of the intervention. The survey included items on food acceptability, consumption of nonstudy food, self-reported compliance, convenience of meal delivery, likelihood of continuing a similar dietary pattern, and willingness to recommend the study to others. These responses captured key dimensions of participant satisfaction, adherence, and the potential for longer-term dietary behavior change. In addition, participants completed daily food checklists throughout the intervention to objectively monitor intake and adherence; details of the checklist design and data collection procedures are described in the methods article [38].

### Sample collection

Overnight fasted blood samples were collected at baseline and at the end of each dietary phase using standard venipuncture procedures. Blood was drawn into red-top tubes for serum and heparinized green-top tubes for plasma. Samples were anonymized, labeled, and immediately processed. Fresh blood was used for routine clinical chemistry analyses, including glucose and lipid panels. Serum was aliquoted and stored at –80°C for later batch analysis of circulating biomarkers, including biogenic amines and neuroactive metabolites. All procedures followed standardized protocols to ensure sample integrity and consistency across time points.

### Biomarker assessment

Cognitive-related metabolic biomarkers were evaluated using fasting serum samples. TC, HDL, triglycerides, and glucose were measured via point-of-care testing using the Cholestech LDX System (Abbott Laboratories), in accordance with the manufacturer guidelines. Serum concentrations of insulin, ferritin, and BDNF were quantified using previously optimized magnetic bead-based multiplex immunoassays (MAGPIX, R&D system Luminex), with BDNF and ferritin analyzed together within the same panel, and insulin measured separately using an individual assay (MAGPIX, R&D system Luminex). These biomarkers are relevant to the health of older adults given their roles in CVD, IR, iron stores, neuroinflammation, and neuroplasticity, all of which are increasingly recognized as interconnected factors influencing cognitive aging. Phosphatidylcholine plays a key role in membrane integrity, lipid metabolism, and neurotransmitter synthesis, making it relevant to both metabolic and cognitive health. Phosphatidylcholine and free choline levels were assessed using enzymatic colorimetric assays (Abcam). Serum homocysteine, involved in one-carbon metabolism and associated with increased risk of CVD and cognitive decline, was measured using the Centaur CP system (Siemens). Batch analyses were performed at the Clinical and Laboratory Services for the Advancement of Science Laboratory at the University of Michigan.

Following our previously published protocols [26], biogenic amine profiling was conducted at the West Coast Metabolomics Center (University of California, Davis) using an untargeted HILIC-qTOF-MS platform. Serum samples were extracted, dried, and reconstituted before assaying biogenic amine metabolites, including kynurenine, GABA, tyrosine, tryptophan, phenylalanine, glycine, and glutamic acid. Injections were onto a Waters Acquity BEH Amide column (1.7 μm, 2.1 × 150 mm) and separation was achieved using a gradient of liquid chromatography–mass spectrometry grade water and acetonitrile with ammonium formate and formic acid. Mass spectra were acquired in positive ion mode with high resolution, and metabolite identification was based on retention time and *m*/*z* values, verified against authentic internal standards. Quantification was performed using external standard curves and internal isotope-labeled controls. Data were processed with mzMine and Agilent MassHunter, and normalized using the Systematic Error Removal using Random Forest algorithm. Final values were reported as calibrated relative intensities to reflect relative abundances.

### CVD risk assessment

BMI was calculated as weight in kilograms divided by height in meters squared (kg/m^2^). Atherosclerotic cardiovascular disease (ASCVD) 10-y risk and estimated vascular age were both calculated using the Framingham Risk Score algorithm, which incorporates age, sex, TC, HDL, systolic blood pressure (SBP), treatment for hypertension, smoking status, and diabetes status. Vascular age was used as a surrogate marker for the biological burden of cardiometabolic risk [41]. SPISE was used to estimate insulin sensitivity and calculated as SPISE = 600 × HDL^0.185^/TG^0.2^ × BMI^1.338^[[42], [43], [44]]. Indices were calculated using fasting values collected at baseline and postintervention time points.

### Anthropometry, body composition, muscle function, and blood pressure

Anthropometric, body composition, and physical function measurements were conducted at baseline and the end of each dietary intervention phase using standardized protocols. Standing height was measured to the nearest 0.5 cm using a wall-mounted stadiometer (Seca), and body weight was recorded to the nearest 0.1 kg using a calibrated digital scale (Seca) with participants in light clothing and no shoes. Body composition was assessed using dual-energy X-ray absorptiometry (Hologic Horizon), which provided estimates of fat-free mass (bone mass + lean mass), referred to as lean mass hereafter. Bone mass is unlikely to change within the short duration of the intervention; hence, any observed change implies lean mass or muscle mass change.

Muscle strength and function were assessed using handgrip strength (measured as the highest value from 3 trials of the dominant hand using a handheld dynamometer) and the 5-repetition chair-rise test (time to complete 5 consecutive unassisted stands from a seated position), where longer completion times indicate poorer lower-body strength and functional performance.

Resting blood pressure was measured at baseline on the upper left arm using an automated sphygmomanometer (GE Carescape V100) after ≥ 5 min of seated rest. Two readings were taken 1 min apart, and the mean was used in the analysis.

### Sample size and statistical analysis

**FIGURE 1:**
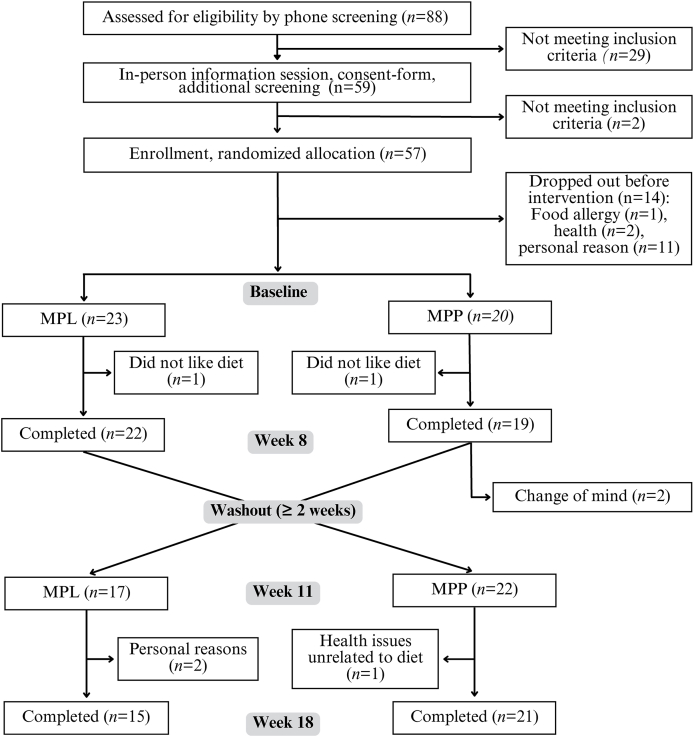
CONSORT flow diagram. MPL, minimally processed lentil; MPP, minimally processed pork; PRODMED2, Protein-Distinct Macronutrient-Equivalent Diet 2.

Most research on red meat fails to differentiate between processed and unprocessed forms. To address our hypothesis that incorporating minimally processed lean red meat within a healthy plant-forward diet would support cognitive-related metabolic health, we proposed markers of insulin sensitivity (insulin), cognitive decline (homocysteine), iron stores (ferritin), as well as muscular fitness (chair stand performance). Sample size calculations for the PRODMED2 trial were based on the ability to detect a clinically meaningful difference in circulating homocysteine, which exhibited the greatest variability in effect size among the primary endpoints based on previously published data: homocysteine, insulin, ferritin, and chair stand performance. On the basis of this, a sample size of 12 participants per diet arm was estimated to provide 90% power to detect the expected difference at a 2-sided alpha level of 0.05 (total probability of making a false positive type I error is 5%, split equally between both tails). To account for an anticipated overall attrition rate of ∼ 25%, the target enrollment was increased to 15 participants per arm (*n* = 30 total). However, due to early observations, we intentionally exceeded the proposed enrollment target to ensure adequate statistical power at study completion. Against an anticipated attrition of 25% throughout the entire study, an unexpected 25% postenrollment dropout before intervention start raised concerns about potential compliance (Figure 1). Additional dropouts or protocol nonadherence were anticipated, particularly given that the vegetarian feeding phase (control intervention) was culturally atypical for the Midwestern older adult cohort. Because of the 18-wk intervention length and these uncertainties, rolling recruitment continued until ≥12 participants in each arm had completed the study. By that point, 43 participants had already begun the intervention, and without a valid reason, we did not wish to remove them from the study.

All statistical analyses were performed using R version 4.3.2 (R Foundation for Statistical Computing) in RStudio, with the overarching statistical analysis framework prespecified in the trial registration. The normality of baseline variables was assessed using the Shapiro-Wilk test. Normally distributed variables were summarized as means ± SDs and compared using unpaired *t*-tests, whereas nonnormally distributed variables were analyzed using Wilcoxon rank-sum tests. Descriptive and inferential analyses for primary and secondary outcomes were conducted using robust linear mixed-effects models implemented with the robustlmm package; models included participant ID as a random effect and fixed effects for timepoint, age, and sex. Least-squares means and 95% confidence intervals were estimated to evaluate biomarker differences across dietary phases. Pairwise comparisons were performed using the emmeans package with Tukey adjustment for multiple testing. Analyses of secondary outcomes were exploratory and hypothesis generating. All tests were 2-sided, and statistical significance was set at *P* < 0.05. For correlation analyses, Pearson’s or Spearman's methods were used as appropriate. Visuals were generated using BioRender (BioRender.com), Adobe Illustrator 2025, Canva (www.canva.com), and RStudio.

## Results

### Participant characteristics

Of the 88 individuals screened, 57 met the eligibility criteria and were randomly assigned to either MPP or MPL (Figure 1). Fourteen participants withdrew prior to baseline assessments, mostly due to personal reasons (scheduling conflicts, obligations) or health-related exclusions. Among the 43 individuals who initiated the intervention (MPP: *n*= 20; MPL: *n* = 23), 7 withdrew during the study period (MPP: *n* = 5; MPL: *n* = 2). A total of 36 participants who were initially assigned to MPP (*n* = 15) or MPL (*n* = 21) completed the full protocol and were included in the final analysis (Figure 1).

Baseline characteristics of participants are summarized in Table 1. The cohort was Caucasian older adults, with 72% female representation. Mean age was similar between sexes (females: 71.7 y; males: 71.8 y). Educational attainment was high, with over 70% of both males and females reporting a 4-y college degree or higher. Nearly half of the participants were retired, and over half were married or living with a partner. Males were more likely to be married than females (80% compared with 50%).

**TABLE 1:** Baseline characteristics of the study participants by sex

Features	Female (*n* = 26)	Male (*n* = 10)
Age (y)	71.7 ± 4.7	71.8 ± 7.0
Race: Caucasian (%)	26 (100)	10 (100)
4-y college educated (%)	19 (73)	8 (80)
Employment status		
Retired (%)	13 (50)	4 (40)
Employed (%)	13 (50)	6 (60)
Current smoker (%)	0	0
Marital status		
Married (%)	13 (50)	8 (80)
Other marital status^1^ (%)	13 (50)	2 (20)
Weight (kg)	74.0 ± 17.0	97.7 ± 16.5
Height (m)	1.65 ± 0.1	1.80 ± 0.1
BMI (kg/m^2^)	27.1 ± 5.7	30.2 ± 4.2
SBP (mm Hg)	128.0 ± 15.6	135.5 ± 18.0
DBP (mm Hg)	71.1 ± 10.1	71.1 ± 14.0
HbA1c (%)	5.4 ± 0.4	5.5 ± 0.2
Vascular age (y)	68.4 ± 11.3	76.9 ± 7.0
10-y ASCVD risk (%)	8.0 ± 2.6	10.78 ± 4.0

Data presented as mean ± SD for continuous variables and as absolute number (%) for categorical variables. Group comparisons between females (*n* = 26) and males (*n* = 10) were conducted using unpaired *t*-tests or Wilcoxon rank-sum tests, as appropriate. Although *P* values are not shown in the table, statistical tests were performed for all variables; only vascular age showed a statistically significant difference (*P* < 0.05).

ASCVD, atherosclerotic cardiovascular disease; DBP, diastolic blood pressure; HbA1c, hemoglobin A1c; SBP, systolic blood pressure.

1 Includes participants who were single, divorced, or widowed.

Participants demonstrated overall good health for their age, free of major chronic diseases but at risk for age-associated cognitive-related metabolic decline. Glycated hemoglobin values were within the normal range for both sexes (females: 5.4% ± 0.4%; males: 5.5% ± 0.2%). SBP was slightly higher in males, whereas diastolic pressure was similar between sexes. Mean BMI classified females as overweight (27.1 ± 5.7 kg/m^2^) and males as obese (30.2 ± 4.2 kg/m^2^). Despite identical chronological age, vascular age was significantly higher in males (76.9 ± 7 y) than in females (68.4 ± 11.3 y; *P* = 0.019). The estimated 10-y ASCVD risk score remained below 11% in both sexes. A comparison of baseline characteristics between completers and dropouts is provided in Supplementary Table 2.

### Nutritional characteristics and compliance of provided diets

All meals were fully provided, promoting consistent dietary intake across participants. Energy levels were similar between intervention phases, ranging from 1982.6 kcal at baseline to 2068.3 kcal in MPP and 2021.8 kcal in MPL (Table 2) with additional details on actual food intake presented in Supplementary Table 3. Protein content comprised ∼ 17%–18% of total energy. Compared with baseline, both diets provided similar energy, lower fats and saturated fats, and increased carbohydrates typical of a plant-forward dietary pattern. The amount of red meat provided increased relative to baseline habitual intake (96 g/d at baseline compared with 162 g/d during the MPP phase), whereas the amount of lentils provided increased from 16 g/d at baseline to 332 g/d during the MPL phase, reflecting the primary protein distinction between the 2 diets. The provided diets met the recommended dietary allowance (RDA) for B vitamins, including B6, B9 (folate), Iron, and B12 (Table 2).

**TABLE 2:** Nutrient composition of the diet phases

Components	Baseline	MPP	MPL
Energy (kcal/d)	1982.6	2068.3	2021.8
Protein (%E)	17.2	18.0	17.2
Carbohydrate (%E)	43.2	55.7	54.2
Fat (%E)	41.6	29.4	30.7
Saturated fat (%E)	12.9	6.8	8.7
PUFA (g/d)	15.2	21.2	17.7
MUFA (g/d)	33.5	12.4	13.8
Sodium (mg/d)	2548.5	2117.6	2194.9
Vitamin B6 (mg/d)	1.6	3.34	1.9
Folate (μg DFE/d)	335.5	489.4	456.5
Vitamin B12 (μg/d)	10.3	2.4	2.6
Choline (mg/d)	316.3	361.6	260.3
Iron (mg/d)	13.7	18.4	18.3
Red meat (g/d)	96.1	162	0
Lentils (g/d)	15.6	0	331.6

Baseline data (*n =* 35) are presented as mean daily energy and macronutrient intake, along with the provided values during the MPP and MPL diet phases. Macronutrient percentage distribution (%E) was calculated based on total energy provided.

MPL, minimally processed lentil; MPP, minimally processed lean pork; %E, percentage of total energy; DFE, dietary folate equivalents.

**FIGURE 2:**
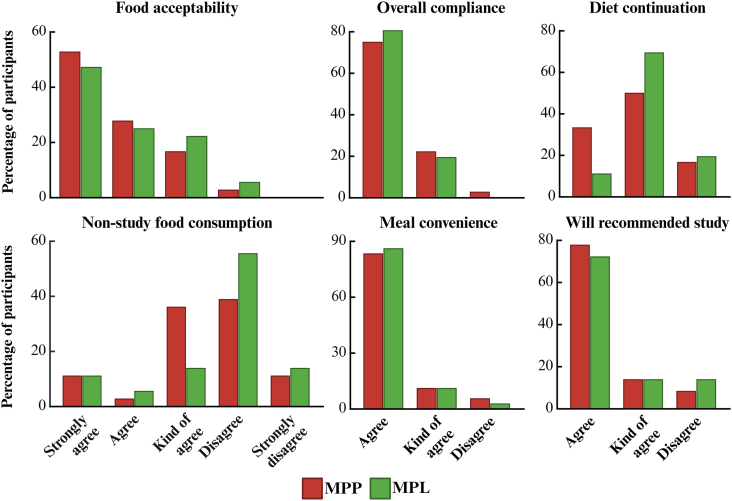
Participant satisfaction and compliance across diet phases. Responses are shown as the percentage of participants in each response category. MPL, minimally processed lentil; MPP, minimally processed pork.

Self-reported adherence and satisfaction were high (Figure 2). The reported 75% refers specifically to participants who strongly agreed with the compliance statement. When combined with those who agreed, overall adherence exceeded 95% across both dietary phases. The convenience of preportioned meals was appreciated (MPP: 83%; MPL: 86%) and >50% of participants in both diets reported no consumption of nonstudy food. A majority indicated interest in continuing a DGA-aligned diet postintervention. More MPP participants (78%) reported they would recommend the study, compared with 72% in the MPL group. Greater cultural familiarity with an omnivorous lifestyle in the Midwest and among this older adult population may have contributed to this survey outcome. Data from daily food checklists, summarized in Supplementary Table 3, further confirms this pattern of high adherence, indicating minimal intake of nonstudy foods during both dietary phases.

### Cognitive-related metabolic biomarker changes

Both diets resulted in favorable changes in cognitive-related metabolic health markers relative to baseline, though the magnitude of response varied across markers (Table 3). Glucose declined significantly in MPL (−5.1 mg/dL, *P* = 0.002), with a similar but nonsignificant reduction in MPP (−3.4 mg/dL, *P* = 0.087). Fasting insulin concentrations decreased in both diet phases (both, *P* < 0.001), suggesting improved insulin sensitivity across both plant-forward diets irrespective of primary protein source. No significant between-group differences were observed for either glucose or insulin (*P* > 0.05). Insulin sensitivity was further assessed using the SPISE index, a noninsulin-based surrogate that incorporates lipid parameters. SPISE increased significantly after the MPP phase (*P =* 0.032), but not MPL (*P* = 0.269), suggesting a possible diet-specific enhancement in metabolic efficiency.

**TABLE 3:** Cognitive-related metabolic biomarker responses across diet phases

Biomarkers	Baseline	MPP	MPP, *P*	MPL	MPL, *P*	MPP vs MPL*,P*^1^
TC (mg/dL)	185 (173, 196)	164 (152, 176)	<0.001	158 (146, 170)	<0.001	0.396
HDL (mg/dL)	54.1 (49.3, 58.8)	44.8 (40, 49.5)	<0.001	40.9 (36.1, 45.7)	<0.001	0.034
Triglyceride (mg/dL)	95.2 (81.9, 108.5)	86.2 (72.9, 99.5)	0.213	88.0 (74.7, 101.3)	0.408	0.980
Glucose (mg/dL)	93.6 (90.5, 96.8)	90.2 (87.1, 93.4)	0.087	88.5 (85.3, 91.7)	0.002	0.641
Insulin (μU/mL)	9.8 (8.6, 10.9)	7.6 (6.4, 8.7)	<0.001	7.9 (6.7, 9)	<0.001	0.765
SPISE Index	5.8 (5.01, 6.56)	6.1 (5.3, 6.8)	0.032	5.98 (5.2, 6.7)	0.269	0.792

Data (*n = 36*) presented as least-squares means and 95% confidence intervals, derived from a robust linear mixed-effects model adjusted for diet phase, age, and sex, with participant ID included as a random effect.

MPL, minimally processed lentil; MPP, minimally processed lean pork; SPISE, Single Point Insulin Sensitivity Estimator; TC, total cholesterol.

1 *P* values for within- and between-group comparisons were obtained from the same model.

Lipid profiles improved under both dietary conditions. TC decreased significantly in both MPP (−21 mg/dL) and MPL (−27 mg/dL) phases (*P* < 0.001), with no significant difference between groups (*P* > 0.05). HDL declined across both diets (*P* < 0.001); however, the reduction was smaller in the MPP group (*P* = 0.034 compared with MPL). Triglyceride levels reduced by a small margin in both groups, but the reductions were statistically nonsignificant. These results demonstrate that both primary protein sources improved multiple metrics of metabolic functions known to be associated with cognitive well-being in aging.

### Neurotransmitter and methylation pathway-related biomarkers

BDNF increased modestly after MPL (*P* = 0.056) and showed no change in MPP, suggesting no adverse effect of pork on circulating BDNF levels. Serum choline was stable across both diet phases despite lower dietary supply, indicating effective physiological regulation of this essential nutrient (Tables 2 and 4). In contrast, phosphatidylcholine levels declined significantly from baseline in both MPP (*P =* 0.002) and MPL (*P* < 0.001), with no significant difference between groups (*P* = 0.802).

**TABLE 4:** Effects of interventions on blood biomarkers of nutrition and neurotransmitter-related compounds

Biomarkers	Baseline	MPP	MPP, *P*	MPL	MPL, *P*	MPP vs MPL, *P*^1^
BDNF (ng/mL)	17.5 (15.1, 19.9)	18.3 (15.9, 20.6)	0.810	20.4 (18, 22.7)	0.056	0.213
PC (μmol/L)	1855 (1725, 1984)	1550 (1421, 1680)	0.002	1494 (1364, 1623)	<0.001	0.802
Choline (μmol/L)	4.7 (4.3, 5.2)	4.5 (4.1, 5)	0.841	4.9 (4.5, 5.4)	0.800	0.456
Hcy (μmol/L)	15.7 (13.8, 17.6)	17.4 (15.5, 19.2)	0.007	15.8 (13.9, 17.6)	0.996	0.009
High Hcy responders^2^	20.5 (18.9, 22.1)	26.2 (24.6, 27.8)	<0.001	20.0 (18.4, 21.6)	0.754	<0.001
Typical Hcy responders^2^	14.8 (13.1, 16.5)	15.7 (14.0, 17.5)	0.208	15.0 (13.2, 16.7)	0.939	0.363
Ferritin (ng/mL)	72.2 (49, 95.4)	80.3 (57.1, 103.5)	0.073	88.2 (65, 111.4)	<0.001	0.084
Kynurenine (RI)	1308.7 (1187.8, 1429.7)	1362.9 (1241.9, 1483.8)	0.772	1253.5 (1132.5, 1374.4)	0.764	0.349
GABA (RI)	769.6 (664.7, 874.5)	976.9 (872, 1081.8)	<0.001	1077.9 (972.9, 1182.8)	<0.001	0.163
Tyrosine (RI)	3852.8 (3487.1, 4218.2)	3967.1 (3601.4, 4322.8)	0.901	3939.6 (3573.9, 4305.3)	0.942	0.994
Tryptophan (RI)	22,108.3 (19,831.6, 24,385)	25,499.4 (23,222.8, 27,776.1)	0.044	27,067.5 (24,790.9, 29,344.2)	0.001	0.511
Phenylalanine (RI)	41,563.5 (38,410.7, 44,716.2)	47,226.4 (44,073.7, 50,379.1)	0.034	49,910.7 (46,758, 53,063.4)	<0.001	0.465
Glycine (RI)	343.4 (293.9, 392.8)	400.3 (350.8, 449.8)	0.010	429.1 (279.7, 478.6)	<0.001	0.305
Glutamic acid (RI)	18,544.9 (16,336.9, 20,752.9)	15,213.7 (13,005.7, 17,421.7)	0.012	15,606.5 (13,398.5, 17,814.5)	0.033	0.940

Data (*n* = 36*)* presented as least-squares means and 95% CIs, derived from a robust linear mixed-effects model adjusted for diet phase, age, and sex, with participant ID included as a random effect.

BDNF, brain-derived neurotrophic factor; CIs, confidence intervals; GABA, gamma-aminobutyric acid; Hcy, homocysteine; MPP, minimally processed lean pork; MPL, minimally processed lentil; PC, phosphatidylcholine; RI, relative intensity.

1 *P* values for within- and between-group comparisons were obtained from the same model.

2 Participants classified as high Hcy responders (*n* = 6) met the following criteria: *1*) baseline homocysteine level >15 μmol/L, *2*) a substantial increase in response to the MPP diet(∼ ≥4 μmol/L), and *3*) minimal or no increase in response to the MPL diet. The remaining participants were classified as typical Hcy responders (*n* = 30). Metabolites are reported as relative intensities, based on normalized peak area values obtained from mass spectrometry analysis.

Homocysteine increased modestly after the MPP phase (+1.7 μmol/L, *P* = 0.007), whereas it remained unchanged during the MPL phase (*P* = 0.996), resulting in a between-group difference (*P =* 0.009) (Table 4). Although homocysteine levels tend to be higher in older adults compared with younger individuals, the commonly accepted threshold for elevated homocysteine remains ∼15 μmol/L across all age groups [45]. Using this threshold, half of the participants (*n* = 18) exhibited >15 μmol/L baseline homocysteine levels. Among these, 6 participants (16.6%) showed a markedly different response to the MPP intervention compared with MPL: their homocysteine levels remained stable after MPL (*P* = 0.754) but rose substantially after MPP (+5.7 μmol/L from an already high baseline of 20.5 μmol/L, baseline to post-MPP, *P* ≤ 0.001; post-MPL compared with post-MPP, *P* ≤ 0.001). For the remaining participants (*n* = 30), the mean baseline homocysteine level was 14.8 μmol/L and remained stable after both MPP and MPL diets (baseline to post-MPL, *P* = 0.939; baseline to post-MPP, *P* = 0.208; post-MPL compared with post-MPP, *P* = 0.363). Homocysteine levels in the 6 hyper-responders to MPP remained substantially higher than in the other 30 participants at all time points (all *P* ≤ 0.004), regardless of diet. Serum vitamin B12 levels were 22%–33% lower in the hyper-responders than in the rest of the cohort at all time points, reaching statistical significance only after MPP (*P* = 0.041), when homocysteine levels were highest. However, all participants consistently maintained serum vitamin B12 concentrations within the clinical reference range. The published reference range of serum vitamin B12 for adults is generally 160–950 pg/mL [46].

Ferritin levels rose in both groups (+8.1 ng/mL post-MPP compared with +16 ng/mL post-MPL) reaching statistical significance in MPL (*P* < 0.001) and trending toward significance in MPP (*P* = 0.073), with no significant difference between diets (*P* = 0.084) (Table 4).

Several neuroactive metabolites were also responsive to dietary intervention. Both groups exhibited significant increases in GABA (MPP: *P* < 0.001; MPL: *P* < 0.001), alongside corresponding reductions in glutamic acid, its excitatory precursor (MPP: *P* = 0.012; MPL: *P* = 0.033), with no between-group differences (*P =* 0.940). These changes may reflect a shift toward greater inhibitory neurotransmitter activity, potentially supporting neuronal homeostasis.

Within the serotonin pathway, tryptophan levels increased significantly in both groups (MPP: *P* = 0.044; MPL: *P =* 0.001), whereas kynurenine remained unchanged. Phenylalanine and tyrosine, both precursors in dopamine biosynthesis, increased, with phenylalanine reaching significance in both groups (MPP: *P* = 0.034; MPL: *P* < 0.001). Glycine, a co-agonist at N-methyl-D-aspartic acid (NMDA) receptors that mediate excitatory neurotransmission, increased significantly in both phases (MPP: *P* = 0.010; MPL: *P* < 0.001) (Table 4).

### Changes in body composition and physical function

**FIGURE 3:**
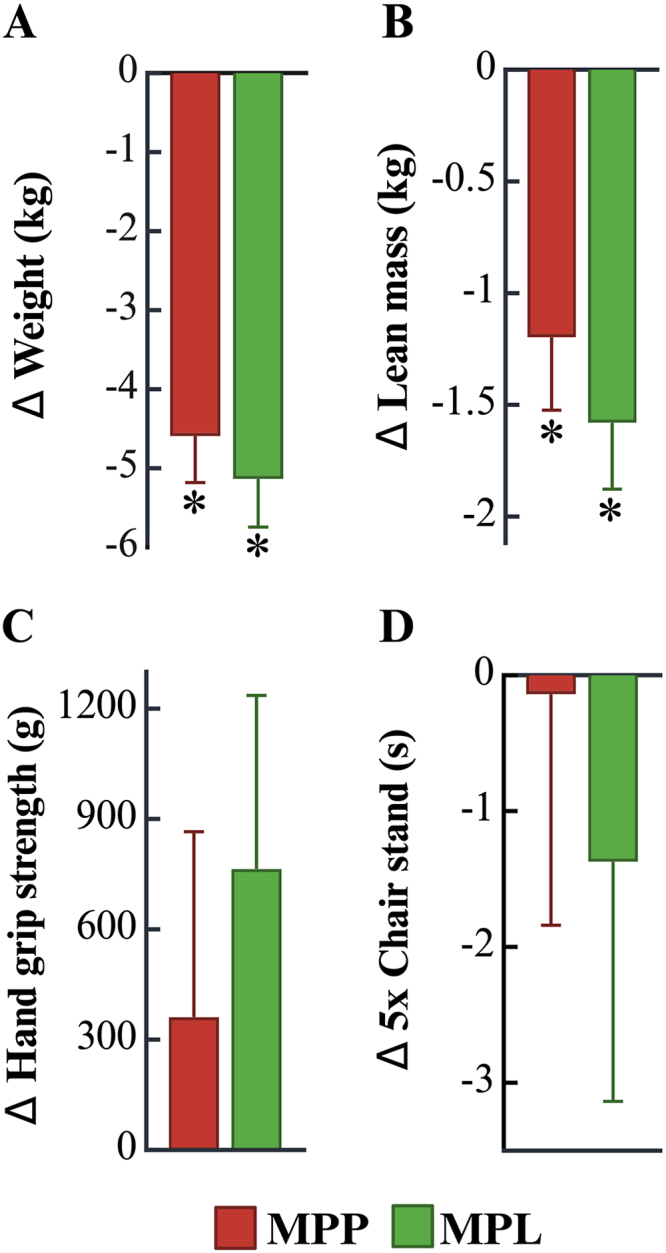
Changes in body composition and physical function across diet phases. Changes in body weight (A), lean mass (B), handgrip strength (C), and chair stand performance (D) across baseline and intervention diets. Change data are presented as mean ± SEM (*n* = 36 for all outcomes, except end-of-study body weight, *n* = 35; chair stand performance, *n* = 32 due to 4 participants being unable to perform the test). Asterisks (∗) indicate significant within-group changes from baseline (*P* < 0.05), based on robust linear mixed models. MPL, minimally processed lentil; MPP, minimally processed lean pork.

As shown in Figure 3A, total body weight significantly decreased after both MPP and MPL diet phases compared with baseline (MPP: –4.6 kg; MPL: –5.1kg; both *P* < 0.001), with no significant difference between diets (*P* > 0.05). With weight loss, lean mass loss may be unavoidable without a substantial strength training intervention, which was not included in this study. Lean mass decreased after both diets (MPP: –1.2 kg; MPL –1.6 kg, both *P* < 0.001) with no statistically significant difference between the diets (*P* = 0.249; Figure 3B).

Functional outcomes are presented in Figure 3C and D. Handgrip strength remained stable across all time points, with minimal improvements but no decline from baseline (*P* > 0.05, between groups). Lower-body function, assessed via the 5-repetition chair stand test, also remained stable after both interventions with no signs of decline (all, *P* > 0.05).

## Discussion

As the number of older adults in the population continues to increase, metabolic-associated neurodegenerative diseases are becoming increasingly prevalent. This randomized controlled feeding intervention evaluated the effects of incorporating minimally processed lean pork daily within a plant-based, nutrient-dense, guidelines-aligned, healthy dietary pattern, which we referred to as plant-forward, on clinical and biomarker-based outcomes related to age- and cognitive-related metabolic functions. In this cohort of older adults with generally healthy baseline profiles for their age, the differences between the presence or absence of red meat in their diet were small. Both dietary interventions led to comparable improvements in insulin sensitivity and other cognitive-related metabolic features, including circulating neuroactive amino acids, whereas functional fitness-related outcomes were preserved without additional strength training intervention. Notably, the absence of adverse health effects from 162 g/d of MPP intake challenges the common perception that red meat intake should be broadly minimized.

At baseline, participants reported typical omnivorous eating habits with moderate red meat intake and minimal lentils consumption; lentil intake averaged <1 serving/wk. The MPP diet increased red meat intake by ∼ 68% compared with baseline, whereas the MPL diet increased lentil intake nearly 20-fold. Therefore, although the MPP phase reflected a moderate adjustment from participants’ habitual diets, the MPL phase represented a more substantial dietary shift toward plant-based proteins in this Midwestern cohort. We also reported a shift from a high ultraprocessed to a whole food dietary pattern during the intervention in our secondary findings [47].

Both diets improved fasting insulin and TC which are independent risk factors for CVD and dementia-related illnesses [48]. The MPP diet notably enhanced the SPISE index, indicating improved insulin sensitivity. Although the primary protein sources differed in essential amino acid (EAA) composition, both diets achieved an EAA-9 score >100, warranting further investigation into the metabolic implications of the amino acid composition differences [38]. Although TC decreased, a modest decline in HDL cholesterol was also observed in both groups, consistent with typical cholesterol-lowering interventions; however, the reduction was smaller in the MPP group. Body weight decreased with both plant-forward diets, and there was a trend toward less lean mass loss after the pork-based intervention. This aligns with prior evidence showing that lean beef intake is positively associated with muscle mass and nutritional status in older adults, suggesting that moderate consumption of lean red meats such as pork may help support muscle maintenance with aging [49]. Although long-term studies are limited, short-term controlled trials indicate that red meat consumption is not harmful and may, in some contexts, be beneficial. For example, observational data often link red meat with adverse metabolic outcomes, but a meta-analysis of 21 RCTs found no consistent negative effects of red meat intake on glycemic or insulinemic risk factors for type 2 diabetes [50]. Similarly, pooled analyses report no significant differences in CVD risk markers when red meat, especially the unprocessed form, was compared with a variety of other dietary proteins [24]. In some cases, red meat consumption has even been associated with improvements in lipid parameters when compared with carbohydrates, mixed animal proteins, or habitual diets [[51], [52], [53]], all of which are in alignment with the observations from this RCT.

Beyond cognitive-related metabolic risk factors sharing pathways between AD and CVD, neurotransmitter dysfunction is a key feature of AD that contributes to both cognitive behavioral symptoms related to age-associated mental health decline. For example, reduced GABA serum levels have been linked to psychological disturbances [54]. In our study, GABA increased, and glutamic acid decreased with both interventions, suggesting a shift toward improved inhibitory balance. The serotonergic system, also disrupted in AD, plays a critical role in memory and mood [55]. Tryptophan, an EAA precursor for serotonin, increased with both diets. But tryptophan’s metabolite, kynurenine, remained unchanged, indicating preserved serotonergic support. For dopamine-related pathways, phenylalanine increased across both diets, whereas tyrosine remained stable, aligning with existing research [56] as potentially beneficial. Additionally, glycine, a coagonist of NMDA receptors, rose modestly and may contribute to enhanced synaptic plasticity and cognitive function [57]. Although not reflective of central levels, these shifts in circulating neuroactive amino acids suggest that a plant-forward dietary pattern may influence peripheral markers associated with brain health. The inclusion of red meat did not diminish these potential benefits in this cohort of older adults. Importantly, these neuroactive biomarker shifts occurred in parallel with favorable cognitive-related metabolic and cardiovascular outcomes, underscoring the need to evaluate red meat within the context of the overall dietary pattern rather than in isolation.

Some studies emphasize the relevance of the dietary matrix and the nature of the comparison diet when assessing red meat’s health effects [58]. Our findings within the context of a plant-forward healthy dietary pattern support the idea that lean, minimally processed red meat can be consumed regularly without adverse cognitive-related metabolic consequences. This has important public health implications, particularly for older adults in rural Midwestern communities where red meat is not only a cultural staple but may also serve as a familiar and acceptable component that facilitates the adoption and long-term adherence to healthier plant-forward dietary patterns. However, feeding studies are typically short duration; longer-term interventions are warranted to determine whether these effects persist over time and to capture potential cognitive changes that may require extended periods to emerge.

Although several outcomes showed clear benefits of red meat intake from baseline to post-diet, other observations were more nuanced, pointing to potential areas of follow-up research. A modest increase in circulating homocysteine was observed in the MPP group but not in the MPL group, when all 36 participants were considered. Homocysteine levels typically increase with age and show an inverse correlation with B12 bioavailability. Intake of vitamin B12, which plays a critical role in homocysteine remethylation and clearance [59], remained above RDA levels during the interventions but was generally lower than baseline intake levels. A study reported that a 9–20 μmol/L blood homocysteine range may be indicative of the lowest mortality rate in elderly patients [60]. The overall increase in homocysteine after MPP was slightly above the reported 16.5 μmol/L physiological range for this age group and well below the 20 μmol/L mark [61]. However, because homocysteine is recognized as a potential marker of cognitive decline [62], we further explored individual variability. Notably, 6 participants with elevated baseline homocysteine levels exhibited a distinct increase in response to red meat intake, unlike the remaining 30 participants whose levels remained stable. This suggests a possible subpopulation effect, which may reflect individual variability in one-carbon metabolism or micronutrient status and warrants further investigation. Studies have shown that some older adults may have a reduced ability to absorb food-bound B12 due to which they exhibit hyperhomocysteinemia in general or in response to higher methionine-containing foods such as red meat [[63], [64], [65], [66]]. The 6 hyper-responders consistently had much lower mean concentrations of serum B12 levels than the remaining 30 participants across all time points. Interestingly, ferritin levels also increased after the MPL phase, despite similar dietary iron content between the 2 diets. This may reflect differences in iron bioavailability or absorption efficiency, which were beyond the scope of the current study but warrant future investigation.

Although the physiological risks and benefits of red meat have been widely studied and debated in general, the specific effects of lean, unprocessed red meat consumed within a healthy dietary matrix on metabolic and neuroactive risk factors remain poorly understood. Short-term randomized feeding trials have generally reported neutral cardiometabolic effects of lean, minimally processed red meat when compared with other high-quality protein sources which aligns with our observations from this research [52,54]. Red meat has been linked to elevated homocysteine, although findings are mixed, with 1 multicenter study reporting that refined cereal consumption was more strongly associated with circulating homocysteine levels than red meat intake [[67], [68], [69]]. On the basis of our homocysteine data, individual differences in baseline Hcy levels underscore the relevance of a precision nutrition approach, suggesting that red meat may have variable effects across individuals. Large-scale and longer-duration studies are needed to clarify whether red meat, when consumed within an overall healthy dietary pattern, supports chronic disease management for most individuals, but may be less suitable for a subset of older adults with distinct metabolic profiles. This view is supported by animal studies showing that altering gut microbiota composition eliminated a diet-induced high-homocysteine phenotype [70]. A larger, adequately powered study is needed to further characterize differences in homocysteine responses to various foods, including red meat, in older adult populations.

Additionally, phosphatidylcholine, a key dietary and membrane-bound source of choline and a precursor for acetylcholine synthesis [71], declined after both dietary interventions. Although free choline levels were maintained, likely due to tight homeostatic regulation, choline intake remained below recommended levels across all time points. The parallel decline in phosphatidylcholine across both groups may reflect a compensatory mechanism [72], rather than a deficiency *per se*. However, this finding raises the possibility of a marginal choline insufficiency, which could be relevant in the context of plant-forward diets for older adults. Although not conclusive, this aligns with existing evidence that choline intake is often suboptimal in the general population [73], and that supplementation may be warranted.

Our study design and implementation protocol had several strengths and general limitations that were separately published [38]. Overall, a feeding study is logistically challenging but offers stronger evidence for causal treatment effects compared with observational studies. However, feeding trials evaluating the effects of minimally processed red meat on cognitive-related metabolic risk factors are especially scarce. Here, we used pork as one of several types of red meat; however, the findings may be broadly applicable to other red meats. The 2 wk washout period is commonly reported in crossover nutrition studies, including our prior work, and was designed to minimize potential carryover effects; however, some residual influence between dietary phases cannot be entirely ruled out [26,74]. A specific limitation of this work relates to the relatively shorter duration of the intervention, due to which functional cognitive testing was not included. We believed that functional cognitive changes would be less likely to emerge within the relatively short 8-wk intervention period. Although the diets were designed for weight maintenance, a modest, unintentional weight loss was observed, which may have contributed to some of the observed metabolic improvements. Another limitation is that the study population consisted exclusively of Caucasian adults, reflecting the demographic makeup of the rural Midwestern region where the research was conducted and representing a group otherwise less frequently included in clinical trials. Although this homogeneity strengthens the internal validity of our observations, caution is warranted when applying these findings to more diverse populations, including individuals of different racial backgrounds, age groups, body compositions, or health conditions.

In conclusion, findings from this randomized controlled crossover feeding trial in rural Midwestern older adults suggest that both plant-forward dietary patterns incorporating minimally processed lean red meat, such as pork, and those emphasizing plant-based proteins, such as lentils, can support cognitive-related metabolic health and may influence peripheral neurochemical markers. Supporting healthy aging may be better achieved through nutrient-dense and balanced diets that draw on high-quality foods from both plant and animal sources. This dietary pattern reflects locally relevant food practices in a rural Midwestern population where red meats and backyard-grown or farm-based plant foods are widely available and commonly consumed. Further research is needed to assess the long-term clinical relevance of these findings, which may also have implications beyond Midwestern rural populations where meat consumption remains culturally prevalent.

## Author contributions

The authors’ responsibilities were as follows – MD: conceived the project, designed the research, and provided resources and study oversight; MD, BOdV, SV, LW: conducted the research and collected data; SV: analyzed and visualized the data; SV, BOdV, MD: contributed to data annotation and interpretation; SV, MD wrote the manuscript and hold primary responsibility for the final content; and all authors: read, helped edit, and approved the final manuscript.

## Data availability

Data are available on reasonable request by contacting the corresponding author.

## Declaration of generative AI and AI-assisted technologies in the writing process

During the preparation of this work, the authors used ChatGPT to assist with sentence structure and grammar. All content was subsequently reviewed and edited by the authors, who take full responsibility for the final version of the manuscript.

## Funding

This work was funded by the National Pork Checkoff (grant #22-038/PR-005310), Meat Foundation (grant #3X4166/SA2400166), and the USDA NIFA/AES (grant #AH831-25). The sponsors had no role in the design of the study; the collection, analysis, or interpretation of data; the writing of the manuscript; or the decision to submit the manuscript for publication. There were no restrictions imposed by the funding sources regarding publication.

## Conflict of interest

The authors report no conflicts of interest.
