# Supplementary material for: Effects of Minimally Processed Red Meat within a Plant-Forward Diet on Biomarkers of Physical and Cognitive Aging: A Randomized Controlled Crossover Feeding Trial
Source: Curr Dev Nutr. 2025 Dec 10;10(1):107615. doi: 10.1016/j.cdnut.2025.107615 (PMC12805104; doi:10.1016/j.cdnut.2025.107615)
Supplement: Multimedia component 1 [file mmc1.docx]

**Supplementary Material**

**Effects of Minimally Processed Red Meat Within a Plant-Forward Diet on Biomarkers of Physical and Cognitive Aging: A Randomized Controlled Crossover Feeding Trial**

**Saba Vaezi^1^, Bruna O. de Vargas^1^, Lee Weidauer^1^, Jessica L. Freeling^2^ and Moul Dey^1^**

**Table S1.** Example Daily Menu for MPP and MPL Diet Phases

|  | MPP | MPL |
| --- | --- | --- |
| Breakfast entrees | Carrot cake baked oatmeal (old fashioned oats) | Carrot cake baked oatmeal (old fashioned oats) |
| Lunch entrees | Maple Chipotle bowl (cubed roasted pork loin, brown rice, spinach, maple syrup, butternut squash, brussels sprouts) | Maple Chipotle bowl (pea protein powder, ground plant meat, chickpea rice, maple syrup, spinach, butternut squash, brussels sprouts) |
| Dinner entrees | Overloaded baked potato (cubed roasted pork loin, potato, cheese, corn, bell pepper, green onion) + Steamed green beans | Overloaded baked potato (pea protein powder, red lentil, potato, cheese, corn, bell pepper, green onion) |
| Snacks and sides | Fruit cup, potato crisp | Fruit cup, yogurt, corn puffs |
| Beverages | ~ 64 oz per day including water, tea, and black coffee, or other zero calorie beverages | |

The menus illustrate the matched meal structure, energy, and macronutrient composition across diets. Minor ingredient differences (e.g., inclusion of chickpea rice in MPL and brown rice in MPP) were implemented to maintain equivalent fiber and nutrient content while preserving a plant-forward, DGA-aligned dietary pattern. Plant meats were sparingly used, while most of the lentils served were cooked from fresh whole lentils.

**Table S2.** Baseline Characteristics of Study Completers and Dropouts

| Variables, n (%) | Completers (n = 36) | Dropouts (n = 7) |
| --- | --- | --- |
| Age (years) | 71.7 ± 5.3 | 71.8 ± 7.0 |
| Sex (m/f) | 10/26 | 3/4 |
| Race: Caucasian | 36 (100) | 7 (100) |
| Four-year college educated | 27 (75) | 2 (28) |
| Current smoker | 0 (0) | 0 (0) |
| Married/Partnered | 21 (58) | 3 (43) |
| Widowed/Divorced | 13 (36) | 3 (43) |
| Single/Never Married | 2 (6) | 1 (14) |
| Weight (kg) | 80.6 ± 19.8 | 84.9 ± 22.4 |
| Height (m) | 1.69 ± 0.09 | 1.65 ± 0.13 |
| BMI (kg/m^2^) | 28 ± 5.4 | 31.31 ± 8.5 |

Values are presented as mean ± SD for continuous variables and n (%) for categorical variables. “Completers” refer to participants who completed both dietary phases, while “Dropouts” refer to participants who withdrew after study initiation.

**Table S3.** Summary of adherence during the MPP and MPL phases

|  | MPP | | | MPL | | |
| --- | --- | --- | --- | --- | --- | --- |
|  | Study foods not consumed (kcal/d) | Extra study foods consumed (kcal/d) | Non study foods consumed | Study foods not consumed (kcal/d) | Extra study foods consumed (kcal/d) | Non study foods consumed |
| Energy (kcal/d) | 535.2 ± 315.57 | 54.84 ± 71.23 | 61.55 ± 91.68 | 560.0 ± 331.16 | 48.0 ± 91.76 | 35.45 ± 46.88 |
| Protein (g/d) | 25.62 ± 15.43 | 1.74 ± 2.41 | 1.74 ± 2.47 | 25.13 ± 15.31 | 1.56 ± 2.98 | 0.94 ± 1.20 |
| Carbohydrate (g/d) | 74.59 ± 44.17 | 7.57 ± 10.18 | 7.69 ± 9.37 | 75.82 ± 45.27 | 6.77 ± 14.16 | 4.90 ± 5.78 |
| Fat (g/d) | 16.73 ± 9.83 | 2.02 ± 2.64 | 2.88 ± 5.69 | 18.56 ± 10.82 | 1.68 ± 2.71 | 1.44 ± 2.71 |
| Fiber (g/d) | 11.25 ± 6.88 | 0.69 ± 1.09 | 0.9 ± 1.06 | 12.18 ± 7.28 | 0.65 ± 1.41 | 0.67 ± 0.84 |
| Sodium (mg/d) | 590.83 ± 339.66 | 27.06 ± 34.99 | 73.05 ± 102.94 | 675.06 ± 382.98 | 27.77 ± 44.28 | 44.63 ± 51.62 |

Values are presented as mean ± standard deviation (SD). *MPP* = minimally processed pork diet; *MPL* = minimally processed lentil diet. “Study foods not consumed” represents energy and nutrients from provided foods that were not eaten; “Extra study foods consumed” reflects additional portions of study foods eaten beyond those prescribed; “Non-study foods consumed” refers to self-reported intake of foods not included in the study menus.
